# Application of platelet-rich plasma and platelet lysate in the treatment of experimental lymphocutaneous sporotrichosi

**DOI:** 10.18502/cmm.5.3.1740

**Published:** 2019-09

**Authors:** Elahe Najafi, Ali Arash Anoushiravani, Nooshin Kalafi, Hamid Reza Mohajerani, Ali Reza Moradabadi, Saman Mortezaeei, Mojtaba Didehdar

**Affiliations:** 1 Department of Microbiology, Islamic Azad University, Arak Branch, Arak, Iran; 2 Department of Internal Medicine, Arak University of Medical Sciences, Arak, Iran; 3 Department of Internal Medicine, Iran University of Medical Sciences, Tehran, Iran; 4 Department of Medical Laboratory Sciences, Arak University of Medical Sciences, Arak, Iran; 5 Infectious Diseases Research Center (IDRC), Department of Medical Parasitology and Mycology, Arak University of Medical Sciences, Arak, Iran

**Keywords:** Animal model, Lymphocutaneous sporotrichosis, Platelet lysate, Platelet-rich plasma, Treatment

## Abstract

**Background and Purpose:**

Sporotrichosis is a subcutaneous and chronic fungal infection that is caused by a dimorphic fungus, namely* Sporothrix schenckii sensu lato*. Lymphocutaneous sporotrichosis is the most clinical form, which accounts for nearly 80% of the cases of cutaneous sporotrichosis. Platelets contain several substances with antimicrobial properties. Regarding this, the present study was performed to investigate the effect of blood-based biomaterials, especially platelets in the treatment of lymphocutaneous sporotrichosis.

**Materials and Methods:**

This study was performed on 12 golden hamsters, divided into three groups of control, platelet-rich plasma, and platelet lysate. For the purpose of the study, *Sporothrix* conidia suspension was injected subcutaneously on the back of the animals. After the induction of subcutaneous lesions, the Gomori methenamine silver method was applied to verify lymphocutaneous sporotrichosis. Subsequently, plasma-rich platelet and platelet lysate were injected into the created lesions in the animals in 3-day intervals (due to the short lifetime of platelets). In the final sage, skin tissue samples were examined to check for the presence of yeast cells and their quantification.

**Results:**

The data were indicative of the presence of yeast cells with/without bud in the tissue of lymphocutaneous sporotrichosis lesions in the infected animals. Histological investigation revealed that each of the two biomaterials under study (i.e., plasma-rich platelet and platelet lysate) played a positive role in the removal of the yeast cells of sporotrichosis.

**Conclusion:**

The results of this study showed that both plasma-rich platelet and platelet lysate were able to effectively prevent from the progression of cutaneous sporotrichosis. Accordingly, much attention has been given to new therapies, including treatment with blood-derived biomaterials.

## Introduction

porotrichosis is a subcutaneous and chronic fungal infection that is caused by a thermodimorphic fungus, namely* Sporothrix schenckii sensu lato *[1]. This infection occurs worldwide; however, it is particularly common in tropical and subtropical regions. The etiological agent of this infection is present as a saprophyte in soil, decaying wood, hay, cornstalks, and sphagnum moss [2]. This infection is usually caused by the traumatic inoculation of the fungi on the skin or mucous membrane during outdoor activities (e.g., farming, gardening, and animal husbandry), as well as roadside injuries [3]. 

The clinical manifestations of sporotrichosis are classified into cutaneous and extracutaneous categories [3]. Cutaneous sporotrichosis is more frequent and presents in three clinical types, including lymphocutaneous, fixed cutaneous, and multifocal cutaneous sporotrichosis [2, 3]. Lymphocutaneous sporotrichosis is the most clinical form of this infection that accounts for nearly 80% of cutaneous sporotrichosis cases [1]. 

The selection of sporotrichosis therapeutic approach depends on the clinical manifestation of the disease and immunological condition of the host. Itraconazole is the drug of choice for the treatment of cutaneous or lymphocutaneous sporotrichosis owing to its effectiveness and safety. Potassium iodide is a therapeutic agent for uncomplicated cutaneous sporotrichosis administered orally at a concentration of 2% [3]. Treatment of both types of sporotrichosis must be continued until ensuring about complete clinical remission to achieve a mycological cure, which is usually attained within 3-6 months [1, 3].

Platelet-rich plasma (PRP) constitutes an autologous concentration of platelets in plasma [4]. Due to the immediate presence of platelets at the site of damage, these cells are the first agents calling out other cells to the site of injury by releasing special mediators [5, 6]. There a number of research addressing the role of platelets in bacterial and parasitic infections [5-7]. Platelet α-granules contain a high concentration of growth factors. These growth factors include platelet-derived growth factor (PDGF), fibroblast growth factor, transforming growth factors (TGF)- β1 and β-2, insulin-like growth factor-1, connective tissue growth factor, platelet-derived epidermal growth factor, platelet factor-4, vascular endothelial growth factor (VEGF), and epidermal growth factor [8-10]. 

The mentioned growth factors facilitate the acceleration of endothelial, epithelial, and epidermal regeneration. Moreover, they account for the stimulation of angiogenesis, enhancement of collagen synthesis and soft tissue healing, and elevation of homeostatic response to an injury [11-14]. Moreover, platelet α-granules contain other substances, such as catecholamines, serotonin, osteonectin, von Willebrand factor, and proaccelerin that may have antibacterial effects [6]. In addition, platelets entail bioactive proteins, such as vitronectin, thrombospondin, and fibronectin causing the attraction of macrophages, mesenchymal stem cells, and osteoblasts [8, 9]. In addition, they enhance tissue regeneration and healing [15-17]. 

Platelets can directly interact with microbial pathogens and eliminate them from the bloodstream. Regarding this, it is considered that platelets contribute to antibody-dependent cell cytotoxicity against microbial pathogens. Platelet microbicidal proteins are released after the activation of platelet against many bacterial and fungal pathogens [18]. The antimicrobial effect of platelets may be increased through their concentration in PRP [6]. 

Nowadays, PRP therapy is applied as a natural alternative to other therapeutic methods, such as surgery, because it is an autologous blood product bearing no risk of transmissible diseases [14-16, 19]. Platelets play a significant role in the regeneration of skin cells, production of collagen and fibroblasts, and angiogenesis within the tissues. Accordingly, currently, PRP and platelet lysate are widely used in the treatment of chronic wounds and reduction of the scars caused by chronic wounds. Moreover, they are adopted for cases subjected to oral or dental surgery [12, 13, 15, 16]. With this background in mind, the present study was performed to investigate the impact of the biomaterials derived from blood, especially platelets, on restraining lymphocutaneous sporotrichosis. 

## Materials and Methods

***Ethics Statement***

This study was approved by the Research Deputy of the Ethics Committee of Arak University of Medical Sciences, Aral, Iran (IR, ARAKMU.REC.1394.364).

***Animals***

The present study was conducted on 12 male golden hamsters (*Mesocricetus auratus*) aged 8-10 weeks and weighing 150-180 g. All hamsters were kept at the animal houses of the Islamic Azad University of Arak, under controlled environmental and nutritional conditions. These animals can be infected with *Sporothrix schenckii*. This infection progresses in their skin lymph nodes and can cause peripherals organ amputation at the end stage. For the purpose of the study, the samples were divided into three groups of control, PRP, and platelet lysate, each of which contained four hamsters. 

Before infecting the animals, the gender of the hamsters was determined by observing milk glands in female rats and the gonads (testes) in male animals. Subsequently, the male animals were selected to be used in the study. Each animal was kept in a separated cage and marked with picric acid. The cage and water bottle had been sterilized in an autoclave before being used in the study. The animals were fed with standard rodent dried food and provided with municipal tap water. The animals were anesthetized using intraperitoneal injections of 50 mg/kg ketamine and 20 mg/kg xylazine administered via the G25 syringes. All procedures were carried out in accordance with the National Institutes of Health Animal Care Guidelines.

***Fungal isolate***

The fungal isolate used in the study was *Sporothrix schenckii* (CBS 356.29). This species was cultured in a Sabouraud dextrose agar medium (Merck, Germany) containing 500 mg/L cycloheximide (Sigma, Germany) and 50 mg/L chloramphenicol (Sigma, Germany) under a sterile condition, and then incubated at 28-30°C for 2 weeks. In the next stage, these colonies were cultured on potato dextrose agar (PDA; Oxoid, Basingstoke, Hampshire, UK) to enhance the conidial product. After the growth of a mature colony on PDA medium at 30°C, suspension was prepared by covering colonies with approximately 8 mL of sterile 0.9% saline. Then, the colonies were gently swabbed with a cotton tip applicator to release conidia from the hyphal. The conidia suspension was withdrawn and transferred to sterile tubes in order to be used for infecting the study animals. 

***Infecting animals***

An area of the skin on the back of the animal that was not accessible to the animal was selected for injection; therefore, the hair was shaved in this area. In order to infect the animals, 0.5 McFarland (1.5×108 approximate conidia suspension) standard conidia suspension was prepared. Subsequently, about 100 µL of standard conidia suspension was injected subcutaneously using a syringe with 25G needle size. In this stage, a swelling resulting from injection appeared on the animal skin. 

All animals were examined for lymphocutaneous lesions every 3 days for 12 weeks. After the induction of lymphocutaneous lesions, a skin biopsy specimen was prepared from one of the infected animals in order to verify the lymphocutaneous sporotrichosis. Biopsy was performed by means of a sterile puncher with a diameter of 4 mm. In the next stage, the obtained samples were transferred to histology laboratory in formalin solution. 

After the preparation of the different sections of the infected tissue, they were stained with Gomori methenamine silver (GMS) and hematoxylin and eosin (H&E) to check for the presence of yeast cells and inflammation, respectively. For confirming the infection, the skin punch biopsies of the lesion were sank in 1 ml of phosphate buffer. After mixture and mechanical digestion, the mixture was cultured in the Sabouraud dextrose agar medium. Infection was indicated by the formation of *Sporothrix schenckii *colony on the Sabouraud dextrose agar after culturing.

***Platelet-rich plasma and plasma lysate preparation***

Expired platelet product bags were used to prepare a PRP sample and platelet lysate. According to the setup of our study, 50 cc of platelet was centrifuged at 1,000 g for 10 min. The supernatant liquid was considered as plasma-rich fragment (PRF). Whole PRF was prepared in a bag as a blood product in the blood bank center. To enrich the PRF, it was centrifuged at 1,500 rpm for 10 min to precipitate the platelets. The reduction of the platelet-poor plasma portion of supernatant resulted in a platelet concentration of 2,500,000 per µl, called platelet-rich plasma (PRP). During the preparation of PRP, the samples were separated using Pasteur pipette, and the count of the platelets in PRP was measured using a cell counter (Sysmex: kx21N, Japan). During this procedure, serial dilution was used to prevent platelet aggregation in the cell counter. 

Platelet lysate was prepared by freezing and thawing platelets to disrupt platelet membrane and release their internal contents. For this purpose, PRP, which was prepared in the prior stage, was stored at -70°C for 30 min, then thawed in a water bath (37°C) for another 30 min. Freezing and thawing stages were repeated for four times. Finally, the suspension was centrifuged at 300 rpm for 15 min to remove the platelet lysate bodies and the supernatant, which was free of the platelets body called “platelet lysate” separated by Pasteur pipette.

***Platelet-rich plasma and platelet lysate injection to the lesion*** ***site***

In order to examine the effect of each biomaterial (i.e., PRO and platelet lysate) on the cutaneous lesions of sporotrichosis, each biomaterial was individually injected inside the cutaneous lesion . The biomaterials were reinjected every 3 days due to the short lifetime of the platelets. Each injection contained 100 µl of biomaterial and was made using a syringe with a needle size of 25G. Every animal was injected four times, and the macroscopic changes of the lesion were examined during that time.

***Microscopic*** ***and*** ***histological*** ***examination of infected tissue***

After the injection of the biomaterials into the sporotrichosis lesions and observing changes in the skin, skin tissue sampling was carried out by a sterile puncher with a diameter of 4 mm to inspect the presence of fungal inflammation. All prepared punches were kept in 10% formalin, transported to the histology laboratory, processed by the tissue processor, and then formed into blocks. Tissue sections with a diameter of 5 µ were cut from blocks and stained by the GMS method after fixation. Subsequently, the prepared slides were examined by a pathologist for the presence and amount of fungus. 

## Results

**Figure 1:**
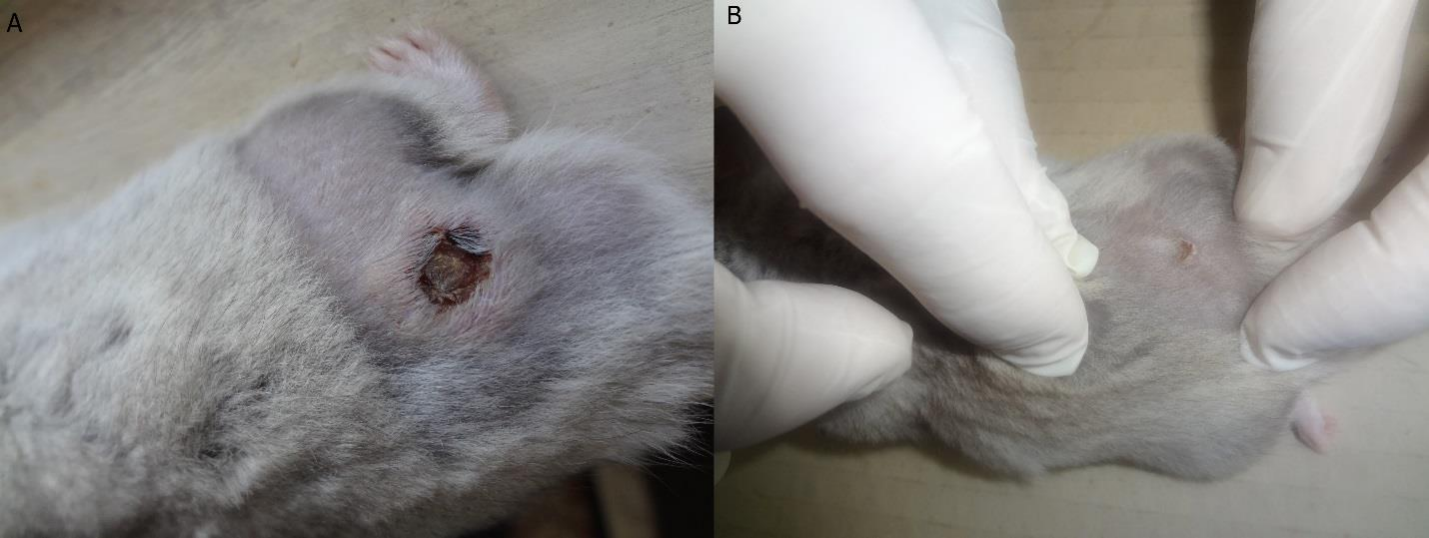
A representative result of the effect of platelet-rich plasma and platelet lysate on lymphocutaneous sporotrichosis lesion in hamsters; a) lymphocutaneous sporotrichosis lesion in a hamster before the injection of plasma-rich platelet and platelet lysate, b) recovery of lymphocutaneous sporotrichosis lesion in hamsters 2 weeks after the injection of plasma-rich platelet and platelet lysate

**Figure 2:**
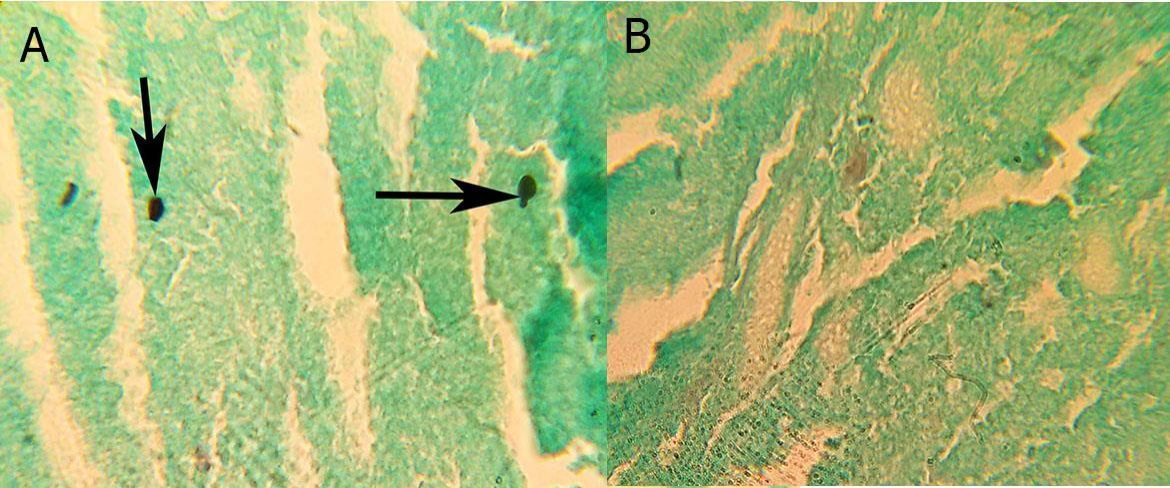
A representative result of the histopathology of lymphocutaneous sporotrichosis tissue in untreated and treated hamsters with plasma-rich platelet and platelet lysate; a) *Sporotrichosis *yeast cells in the histopathology of lymphocutaneous lesions in the untreated hamsters (Gomori methenamine silver stain ×1000), b) removal of the yeast cells in the histopathology of lymphocutaneous sporotrichosis tissue in the treated hamsters (Gomori methenamine silver stain ×400)

**Figure 3:**
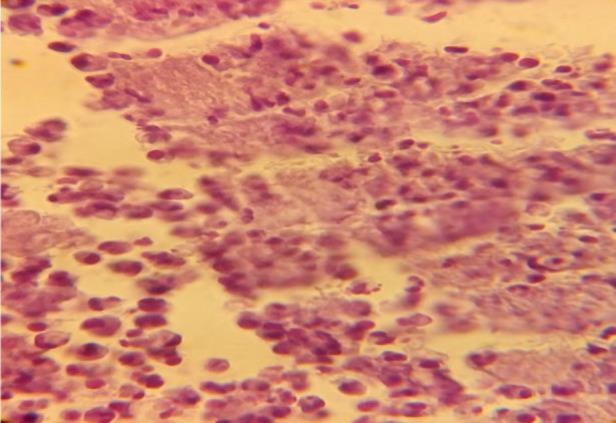
Neutrophil-rich inflammation in the histopathology of lymphocutaneous sporotrichosis tissue (hematoxylin and eosin stain ×400)

Lymphocutaneous sporotrichosis emerged at the site of injection in all three groups after 4 weeks (Figure 1A). Yeast cells with/without bud were observed in the tissue histology of lymphocutaneous lesions in all three groups, as confirmed by a pathologist (Figure 2A). In this stage, the pathologist counted the number of the yeast cells at a mean of 10 within a microscopic field of ×40. In addition, H&E staining revealed neutrophil-rich inflammation in the infected tissue of the hamsters in all three groups (Figure 3).

In the non-treated hamsters, the slides prepared from the tissue of lymphocutaneous lesions showed infection with the yeast cell count of 12.8 in mean 10 of ×40 microscopic field. In the hamsters treated with plasma and lysate, the prepared slides from the tissue of lymphocutaneous lesions showed infection with the yeast cell counts of 5 and < 1 in mean 10 of ×40 microscopic field, respectively. After a week, the lesion size in the non-treated hamsters was measured about 10 mm (Figure 1A). However, this value was obtained as about 5 and < 2 mm in the PRP- and lysate-treated hamsters, respectively. 

**Figure 4:**
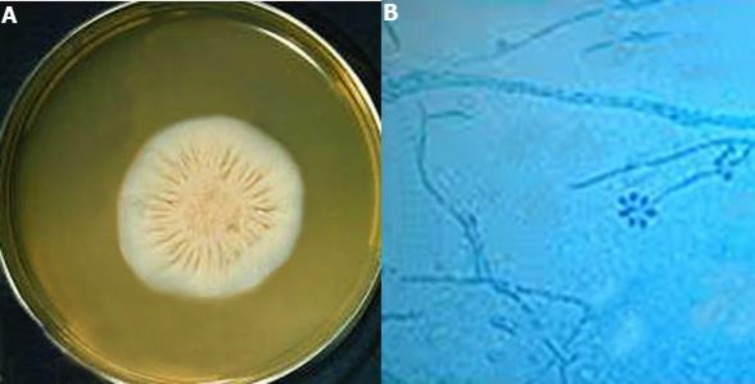
Representative macroscopic and microscopic analyses of *Sporothrix schenckii* colony recovered from lesions; a) *Sporothrix schenckii* colonies on Sabouraud dextrose agar medium growing after 14 days at 30°C, b) microscopic analysis of *Sporothrix schenckii* colony showing delicate branching septate hyphae with slender, short conidiophores with tapering tips and surrounding pyriform conidia in a flower-like arrangement (lactophenol cotton blue stain ×400)

Recovery of *Sporothrix schenckii* from the lesion and the colony formation on the Sabouraud dextrose agar medium were confirmative of lymphocutaneous lesions in the hamsters. The colony was identified using the slide culture method (Figure 4). Two weeks after the injection of PRP and platelet lysates, the lesions were recovered, and no inflammation and secretion were observed (Figure 1B). However, in the control group, there was no change in the lesion and inflammation, and secretion was still observed.

Histological examination revealed no yeast cells after 2 weeks of PRP and platelet lysate injection (Figure 2B). However, these effects occurred more quickly in the group administered with platelet lysate, compared with those in the group subjected to PRP. 

## Discussion

Sporotrichosis is a chronic mycosis that more frequently occurs in cutaneous and subcutaneous forms than in disseminated forms [1]. In this disease, the alternative pathway of the complement system can be activated by C3b component deposition on the fungal cell wall of the yeasts, resulting in the lysis of the yeast cells. In addition, acquired immunity response against the yeasts requires the action of activated macrophages which can be activated by CD4 T lymphocytes during sporotrichosis infection. These macrophages are produced from two different sources, namely the tissue stem cells and blood circulating monocytes migrating to the tissue during inflammation [20].

In the current study, PRP and platelet lysate were able to effectively prevent the progression of lymphocutaneous sporotrichosis. In this regard, the yeast cells disappeared in the infected skin tissue within 2 weeks, and the appearance of the lesions was improved. However, it should be noted that platelet lysate facilitated a shorter recovery period (i.e., within 10-12 days) than PRP.

To the best of our knowledge, this study is the only experimental research addressing the effect of PRP and platelet lysate against fungi. Only Yung et al. and Aggour et al. have investigated the antimicrobial effect of PRP against periodontal pathogens in vitro [18, 21]. The results of both studies were suggestive of the inhibitory role of PRP against bacterial and fungal (*Candida*) growth. Similar to the results of these studies, our finding showed the efficiency of PRP and platelet lysate against *Sporothrix* yeast.

In a study, Goncalves et al. examined the migration mechanism of leukocytes to *Leishmania* infection sites. They demonstrated that monocytes with GR1 markers had a better ability to kill intracellular parasites. They also reported that these cells migrate to the sites of infection when CCL2 cytokine is present [22]. The results of the mentioned study indicated the capability of platelets in causing some cells to selectively migrate to the site of *Leishmania* infection by inducing cytokine production. 

The results of the current study showed that both PRP and platelet lysate were able to effectively prevent the progression of cutaneous sporotrichosis. Moreover, the yeast cells disappeared in the infected skin tissues, and the appearance of lesions was improved within 2 weeks. In addition, Anitua et al., addressing the wound healing ability of platelets, reported that due to the presence of PDGF, TGF-β, and VEGF in platelets, these cells are able to secrete these factors and alter the metabolism of the cells in the secretion site [23]. These alterations can accelerate the process of wound healing. 

In a study conducted by Mazzucco et al., the use of platelet was found to speed up the treatment of refractory wounds [12]. Moreover, in another study investigating diabetic foot wound, Ahmed et al. found that dressing wound with platelet gels had a positive effect [11]. These results are in line with those obtained in the present study. 

In the current study, the results were indicative of the recovery of lymphocutaneous sporotrichosis after the administration of PRP and platelet lysate. In this regard, the macroscopic and microscopic investigation of the wound healing process demonstrated desirable outcome after 2 weeks of using these products. According to the previous studies, these results could be related to platelet healing factors or migration of immune cells to the affected site.

## Conclusion

Modern therapies, including treatment with the blood-based biomaterials, are currently the topics of interest to researchers. In this study, the use of platelet biomaterials for the treatment of lymphocutaneous sporotrichosis rendered favorable therapeutic effects by inducing a considerable decrease in yeast cells. Therefore, it is suggested to apply these biomaterials for the treatment of the patients suffering from this condition.
